# Measurement Properties of the Spinal Function Sort in Patients with Sub-acute Whiplash-Associated Disorders

**DOI:** 10.1007/s10926-014-9559-9

**Published:** 2015-01-21

**Authors:** M. A. Trippolini, P. U. Dijkstra, J. H. B. Geertzen, M. F. Reneman

**Affiliations:** 1 Department of Work Rehabilitation, Rehaklinik Bellikon, Suva Care, 5454 Bellikon, Switzerland; 2 Department of Rehabilitation Medicine, Center for Rehabilitation, University Medical Center Groningen, University of Groningen, Groningen, The Netherlands; 3 Department of Oral and Maxillofacial Surgery, University Medical Center Groningen, University of Groningen, Groningen, The Netherlands

**Keywords:** Whiplash injuries, Neck pain, Physical function, Questionnaires, Disability evaluation, Work

## Abstract

*Purpose* To extensively analyze the measurement properties the Spinal Function Sort (SFS) in patients with sub-acute whiplash-associated disorders (WAD). *Methods* Three-hundred-two patients with WAD were recruited from an outpatient work rehabilitation center. Internal consistency was assessed by Cronbach’s α. Construct validity was tested based on eight a priori hypotheses. Structural validity was measured with principal component analysis (PCA). Test–retest reliability and agreement was evaluated in a sub sample (n = 32) using intraclass correlation coefficient (ICC) and limits of agreement (LoA). The predictive validity of SFS for future work status at 1, 3, 6, and 12 months follow-up was determined by area under the curve (AUC) of receiver operating characteristics. Non-return to work (N-RTW) was defined with two cut-off points: workcapacity <50 and <100 %. *Results* N-RTW decreased from 50 %, 1 month follow-up, to 14 %, 12 months follow-up. Cronbach’s α was 0.98, PCA revealed evidence for unidimensionality. ICC was 0.86, LoA was ±33 points. Seven out of eight hypotheses for construct validity were not rejected. AUC reduced with a longer follow-up from 0.71 for 1 month to 0.61 at 12 months, for cut-off point <50 %. For cut-off point <100 % these values were 0.71 and 0.59. *Conclusion* In patients with sub-acute WAD test–retest reliability, internal consistency, construct- and structural validity of the SFS were adequate. LoA were substantial. Sensitivity to accurately predict N-RTW was poor. The predictive validity of the SFS for N-RTW of patients with sub-acute WAD from an outpatient work rehabilitation setting was only sufficient for the short term (1 month).

## Introduction

Self-report questionnaires have been developed for many types of health conditions, some for use in occupational rehabilitation. One of the reasons for their popularity is the relative efficiency of data collection. In limited time, a broad array of data can be collected about the functional impairments, limitations, and psychological status experienced by the evaluee. This information can be very useful for planning return to work interventions.

However, disability questionnaires have important limitations for use in European occupational rehabilitation settings. The first is that the use of self-reported measures depends on the literacy and linguistic skills of an evaluee which may be limited in evaluees with different cultural backgrounds i.e. mother languages [1]. The second is that most disability instruments do not have a work-related point of reference, but consider an unlimited spectrum of activities. Whether or not the evaluee can actually lift 15 kg at work, for example, is still unknown after filling in the questionnaire. These limitations may be overcome by using a picture-based questionnaire such as the Spinal Function Sort (SFS) [2]. The SFS is a self-report measure of tasks and activities that includes a picture to each item [3]. The items are linked to demonstrable physical ability. The SFS is used in conjunction with a functional capacity evaluation (FCE) to cross-reference self-reported abilities with measured abilities (i.e. functional capacity) [4].

In patients with chronic low back pain (CLBP) the SFS has revealed good clinical practicality, reliability and high predictive validity for non-return to work in various settings and countries [5–8]. Although, the SFS is used in occupational health for other health conditions as well, the measurement properties including the (predictive) validity for future compensation benefits of SFS other than CLBP are unknown. Furthermore, it is not reported whether the SFS performs differently in samples which are assessed earlier in the course of the disorder.

Hence, the aim of this study was to test measurement properties of the SFS by assessing internal consistency, test–retest reliability, agreement, construct validity and predictive validity for work status of the SFS in patients with sub-acute WAD.

## Methods

### Subjects, Procedure and Context

#### Subjects

This study was embedded within usual care of an outpatient work rehabilitation setting. From January 2011 to January 2012 eligible participants were referred for an interdisciplinary rehabilitation assessment at the rehabilitation clinic in Bellikon (Switzerland) by insurance physicians or case managers of Swiss Accident Insurance Fund (SUVA). Participants were from the German-speaking part of Switzerland. The main reasons for referral included: (1) not regaining full work capacity (WC) within 6–12 weeks after a whiplash injury; (2) exceeding expected healing times; (3) or having plateaued with the provided medical and rehabilitative care. Inclusion criteria were: injured workers with WAD related neck pain and, Grade I or II according the Québec Task Force Classification with reduced working capacity of their actual job. They were within 6–12 weeks after initial injury, and received worker’s compensation benefits.

Ethical approval for this study was granted by the Medical Ethics Committee of the Canton Aargau (EK AG 2010/055). Patients gave consent that their data were used for research purpose.

#### Procedure

At base line a review of the medical history and a physical examination was performed by a rehabilitation physician (approximately 60 min), followed by FCE tests administered by a physiotherapist. After determination of eligibility, patients completed questionnaires and carried out FCE tests (60 min). Fitness-for-work certificates or work capacity settlement were explicitly *not* part of this interdisciplinary assessment.

#### Context

All participants were insured by SUVA, the largest state owned accident insurance in Switzerland. SUVA covers costs for occupational and non-occupational injuries for employed individuals and unemployed job-seeking persons [9]. Injured persons receive compensation up to a maximum of 80 % of the previous salary, and medical and vocational assistance. Invalidity pensions can also be refunded by SUVA to the injured person.

### Measures

#### SFS

**Fig. 1:**
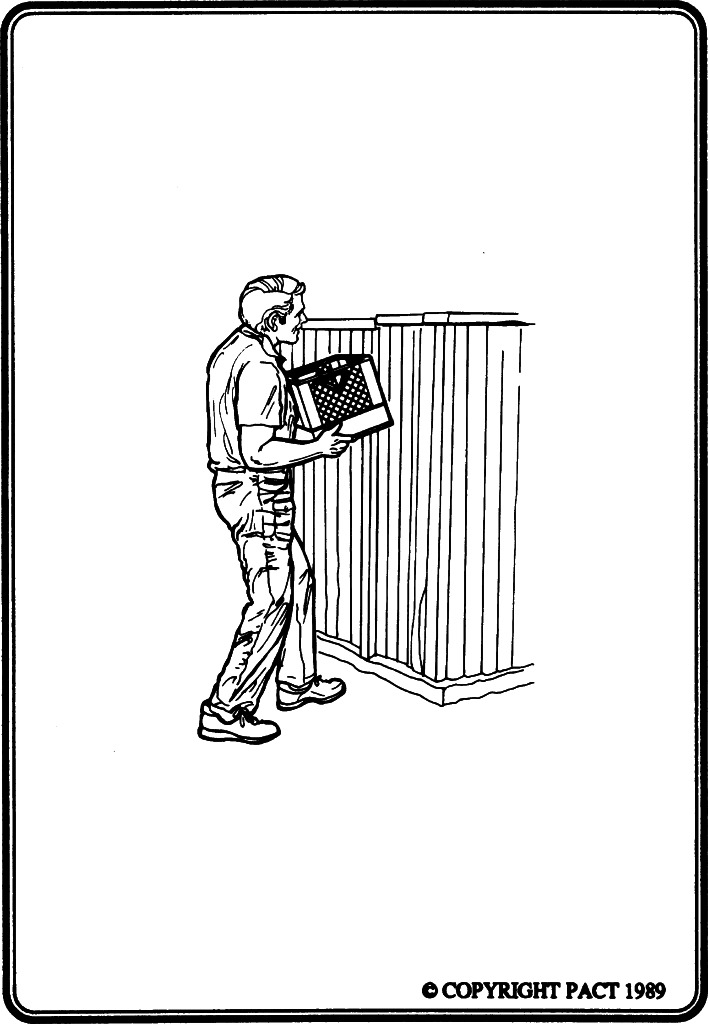
Item 14 of the Spinal Function Sort (SFS) questionnaire: Lift a 10 kg milk crate from the floor to eye-level

The SFS was used to measure self-reported functional ability to perform work-related tasks and activities of daily life that involve the spine. The SFS contains 50 drawings with simple descriptions (Item example in the Fig. 1). Patients rated their functional ability for each activity on a 5-point Likert scale: “able” (4), to “restricted” (1, 2, 3) or “unable” (0). The SFS yields a single rating ranging from 0 to 200, with higher scores indicating more or better abilities. The scores can be categorized according the work demands as defined by the Dictionary of Occupational Titles (DOT) [10]. SFS scores have been adapted to the DOT categories previously as follows [5]: SFS score <100 ≈ minimal work demands, 100–124 ≈ sedentary work (<5 kg), 125–164 ≈ light work (5–10 kg), 165–179 ≈ medium heavy work (10–25 kg), 180–194 ≈ heavy work (25–45), >195 ≈ very heavy work (>45 kg) These categories allow a comparison between the self-reported functional ability and work demand. For test–retest reliability of the SFS a sample of patients was tested twice within a week after baseline.

### Physician Determined Work Capacity (WC)

To determine the predictive validity for future work status, the WC was used as an estimate of ability of work. The WC was obtained from the accident insurance’s administrative data. WC was determined at 1, 3, 6 and 12 months after baseline by the treating physician, usually a general practitioner, and represents the proportion workability of pre-injury work. Determination of WC was based on proposed WC-forms and recommendations [11, 12]. WC is expressed in a percentage (0–100 %) and is translated in days or hours modified work. For example, if a worker is deemed WC = 50 %, he will work for 2.5 days/week or 5 half days/week modified work. The remaining 50 % is financially compensated. The reliability and validity of the WC determination is unknown. WC in %, is directly related to compensation costs and reflects the proportion of work loss to the employer, the employee and the insurance. Therefore, this method of WC-determination may be less dependent to distortion compared self-reported measures of WC [13].

#### FCE

FCE is a standardized battery of functional tests that intend to measure a patient’s safe physical ability for work related activity [14]. For the purpose of this study four lifting tests were analyzed: lifting floor to waist, lifting waist to overhead, short two handed carry, long one-handed carry (right). Patients were asked to perform the test to their maximum ability. The tests have good reliability and acceptable agreement in patients with WAD [15].

#### Pain

Pain intensity was measured with an 11-point numeric rating scale (NRS) ranging from no pain (0) to worst pain (10) [16]. The patient was asked to rate his momentary pain (“pain now”). The NRS is a commonly used scale with proven reliability and validity in patients with neck pain [17].

#### Disability

Neck pain-related disability was measured with the Neck Disability Index (NDI) [18]. The NDI contains 10 items: pain intensity, personal care, lifting, reading, headaches, concentration, work, driving, sleeping, and recreation. The scale of each item ranges from no disability (0) to total disability (5). A higher score indicates more severe self-reported disability. The NDI is reliable and valid in several languages and settings [18, 19].

#### Mental Distress

The Hospital Anxiety and Depression Scale (HADS) was used to assess the symptom severity of anxiety disorders and depression in non-psychiatric populations [21]. The HADS consists of two scales, one for anxiety and one for depression (A- and D-scale respectively). Each scale contains 7 items, with each item rated from 0 (best) to 3 (worst). The scale scores are calculated by summing the responses to the items up to a maximum score of 21 points (severe case) per scale. A higher score indicates more severe anxiety or depression. Good reliability, validity have been reported for the use of the HADS in the general and various clinical populations [20, 21].

### Data Analysis

Normal distribution was visually assessed using P–P plots and tested with the Kolmogorov–Smirnov and the Shapiro–Wilk tests. Floor and ceiling effects for the SFS were considered to be present if more than 15 % of participants achieved the lowest or highest possible score of the items [22].

#### Internal Consistency

Internal consistency was assessed by item-to-total correlations and Cronbach’s alpha. Optimal consistency for measurements at group level was considered when alpha value is between 0.7 and 0.9. Values <0.7 may be indicative for items measuring different traits, values >0.9 may be indicative for item redundancy [23].

#### Unidimensionality

The unidimensionality of the 50 SFS items was measured with principal component analysis (PCA) with Kaiser normalization and Varimax rotation. An Eigenvalue criterion of 1.0 was used for the factor analysis. Unidimensionality was assumed when ratio of the first to the second factor was 3:1 [24].

#### Test–Retest Reliability and Agreement

Test–retest reliability was expressed as an Intraclass Correlation Coefficient (model 1; one-way random) (ICC). ICC was interpreted as follows: ICC ≥ 0.90 is excellent; good when ICC was between 0.75 and 0.90; moderate when ICC was between 0.50 and 0.75; and poor when ICC ≤ 0.50. ICCs were acceptable when ICC ≥ 0.75, and the lower boundary of the 95 % confidence interval of the ICC ≥ 0.50 [25]. Agreement was expressed in limits of agreement (LoA) (mean difference ± 1.96 × SD of mean difference) [26].

#### Construct Validation: Hypothesis Testing

Eight predefined hypothesis on the strength of the association of SFS and four FCE lifting tests, NDI, Pain NRS, and HADS A + D are displayed in Text Box A. The strength of the association is expressed in the absolute value of the correlation coefficient. Associations were calculated using Spearman rank correlation coefficient and interpreted as follows: 0.00–0.25 little if any (“not correlated”); 0.26–0.49 low or weak; 0.50–0.69 moderate; 0.70–0.89 high or strong; 0.90–1.00 very strong correlation [27]. The SFS was considered valid, when 7 out of 8 hypotheses (≥80 %) of the a priori hypotheses were not rejected [28].

**Text Box A:** Eight hypotheses for examining construct validity of the Spinal Function Sort

	Reference test	The validity is not rejected if the strength of the relationship of SFS with	r cut-off values
1	Lifting tests: Lifting floor to waist Lifting waist to overhead Short carry two-handed One-handed carrying right	Functional lifting tests is moderate to high	0.50 ≤ $\left| \text{r} \right|$ ≤ 0.89
2	Self-reported disability (NDI)	Self-reported disability is moderate	0.50 ≤ $\left| \text{r} \right|$ ≤ 0.70
3	Pain now (NRS)	Pain is low or weak	0.25 < $\left| \text{r} \right|$ < 0.50
4	Anxiety (HADS A)	Anxiety is low or weak	0.25 < $\left| \text{r} \right|$ < 0.50
5	Depression (HADS D)	Depression is low or weak	0.25 < $\left| \text{r} \right|$ < 0.50

Lifting tests include lifting floor to waist (kg), lifting waist to overhead (kg),) short carry two-handed (kg), one-handed carrying right (kg). $\left| \text{r} \right|$ = correlation coefficient, absolute value. The direction of the association depends on the scoring of the reference measure

*NRS* Numeric rating scale, *NDI* Neck Disability Questionnaire, *HADS* Hospital Anxiety Depression Scale

#### Predictive Validity for Work Status at 1, 3, 6 and 12 months

Sensitivity and specificity, positive predictive value as well as likelihood ratio of a positive test were calculated to evaluate the predictive validity of the SFS items at baseline for work capacity at 1, 3, 6 and 12 months after baseline assessment. In a setting of injured workers, who are in a transition phase from acute to chronic disorder, the aim is to identify those patients with a high probability of not returning to work (N-RTW) in order to target specific rehabilitation interventions to those patients. We used two cut-off points to measure N-RTW i.e. WC < 50 %, or WC < 100 %. These two cut-off points were determined based on distribution-plots of WC. The index test was the SFS. Sensitivity was defined as the proportion of patients, identified for different DOT categories based on the SFS score, not have N-RTW. Specificity was defined as the proportion of patients, identified for different DOT-categories based on the SFS score, who did return to work. The positive predictive value for N-RTW was calculated as the percentage of patients within a DOT category that were correctly identified not to have regained full work capacity. Likelihood ratio was calculated as Sensitivity/1 − Specificity. Based on a previous study, it was expected that “minimal”, perceived ability (SFS score <100, less than sedentary work) score would have a high positive predictive value in identifying those patients who would N-RTW at follow-up times [5]. Receiver operating characteristic (ROC) curves were drawn and area under the curve (AUC) was calculated. The AUC has a maximum value of 1.0, indicating a perfect predictive validity which is reached if the curve lies in the upper-left corner; a value of 0.5, represented by the diagonal, means that the measurement instrument cannot distinguish between patients N-RTW or RTW. An AUC of at least 0.70 is considered “appropriate” [29]. As a cut off indicating statistical significance *p* < 0.05 was used. All analyses were performed using SPSS (Statistical Package for Social Sciences, Version 21).

## Results

### Patients

From January 2011 to January 2012, 313 subjects were eligible based on the inclusion criteria. Seven SFS scores were missing. In the construct validity study 306 subjects were included. From this sample 302 were included in the study on the predictive validity of the SFS because 4 patients no follow-data on WC were available (Table 1). For the test–retest reliability 32, 11 females, 21 males, mean age 39.6 years, were assessed twice within a week. The patients characteristics of the test–retest study are reported elsewhere [15].

**Table 1:** Characteristics of the patients (n = 302)

Characteristics, unit or scale	
Age (years)	36.1 (11.5)
Female, n (%)	130 (43.0)
Marital status, n (%)	
Married or co-habitation	155 (51.3)
Single	104 (34.4)
Divorced or living separated	41 (13.6)
Other (e.g., widowed)	2 (0.7)
Mother language, n (%)	
Swiss (-German)	157 (52.0)
Albanian	79 (26.2)
Serbo-Croatian	23 (7.6)
Italian	16 (5.3)
Turkish	10 (3.3.)
Arabic	7 (2.3)
Portuguese	3 (1.0)
Spanish	1 (0.3)
Other^a^	6 (2.0)
Duration since WAD injury claim opening (days)^b^	91.0 (72; 125.0)
Attorney involved, n (%)	82 (27.2)
Work status: job contract, n (%)	240 (79.5
Education^c^, n (%)	
Low	142 (47.0)
Intermediate	152 (50.3)
High	8 (2.6)
FCE tests:	
Lifting floor to waist (kg)	19.4 (10.1)
Lifting waist to overhead (kg)	10.7 (5.8)
Short carry two-handed (kg)	23.7 (12.2)
Long carry one handed (kg)	16.9 (7.6)
Self-reported measures (scoring range)	
Pain now (NRS, 0–10)^b^	5.0 (3.0; 6.0)
Perceived functional ability (SFS, 0–200)^b^	141.0 (103.00; 167.0)
Disability (NDI, 0–50)	22.4 (8.3)
Anxiety (HADS A, 0–21)^b^	9.0 (5.0; 12.0)
Depression (HADS D, 0–21)^b^	7.0 (3.0; 10.0)

^a^Other = 1 Polish, 1 Dutch, 1 unknown

^b^If data have a skewed distribution median and an interquartile range, else mean and SD are provided

^c^Level of education: low = no vocational education, intermediate = vocational education, high = bachelor or higher education

### Internal Consistency, Ceiling Effects

Internal consistency was Cronbach’s alpha 0.98. Removing 50 % of the items (even or uneven items), resulted in alpha values of 0.97. Ceiling effects were not present, except in items 45–48. The item to total correlation was <0.20 in item 45–48. These four items displayed very heavy material handling tasks (>45 kg). In a post hoc analysis, Cronbach’s alpha values were unchanged when removing item 45–48. All other items showed item to total correlations >0.30.

### Unidimensionality

Correlations coefficients between each of the SFS were in the majority >0.3. PCA with fixed factors showed the presence of six components with Eigenvalues exceeding 1, explaining 55.3, 8.2, 4.6, 3.2, 2.3 and 2.1 % of the variance, respectively. The inspection of the scree plot revealed 2 components. For the interpretation of the components Varimax rotation was executed. The rotated solution revealed the presence of a mixed structure with two components showing a number of strong loadings. The items 45–48 loaded on a different component. The ratio from the first to the second Eigenvalue was 6.87, indicating reasonable evidence for unidimensionality.

### Test–Retest Reliability and Agreement

**Fig. 2:**
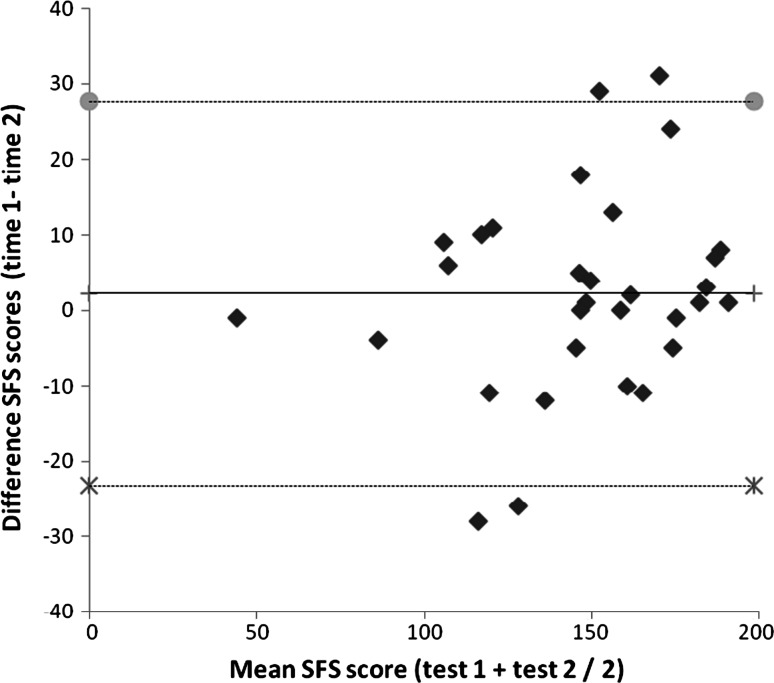
Bland-Altman plot of the SFS scores. The middle line represents the mean difference between the two tests. *Gray* *circle* represent the upper and *cross* *symbol* represent lower limit of agreement, i.e. mean difference + 1.96 SD of the differences and mean difference − 1.96 SD of the differences, respectively. An outlier with a difference in SFS scores of 62 is not shown

The test–retest reliability measured with the ICC was 0.86 (95 %CI 0.71; 0.93). For the 32 patients in the reliability study, mean SFS scores for test and retest were 146.4 (mean, SD 32.1), and 146.6 (mean, SD 37.2) respectively. Mean difference in SFS score between test and retest was 0.2 (SD 16.9, *p* = 0.0.943). Hence LOA were 0.2 ± 33 points Variances were not related to the magnitude of the score. A highly influential patient with a difference of 62 units between tests was detected. LoA calculated without that patient were −23.2 and 27.7 with a mean difference of 2.2 (Fig. 2).

### Construct Validity

#### Construct Validation: Hypothesis Testing

Spearman rank correlations coefficient between the SFS and FCE tests were for lifting floor to waist: 0.68; for lifting waist to overhead: 0.61; for short two-handed horizontal carry: 0.70; for one-handed carry right: 0.64. Correlations between the SFS and disability was −0.62; with pain: −0.49; with anxiety: −0.49 and with depression: −0.52. All correlations were significant (*p* value <0.01). Seven of eight hypotheses were not rejected. Correlations between SFS and work-related lifting tests was moderate to high (0.61–0.70). Depression showed a slightly stronger correlation than hypothesized.

#### Predictive Validity for Work Status at 1, 3, 6 and 12 months Follow-Up

**Fig. 3:**
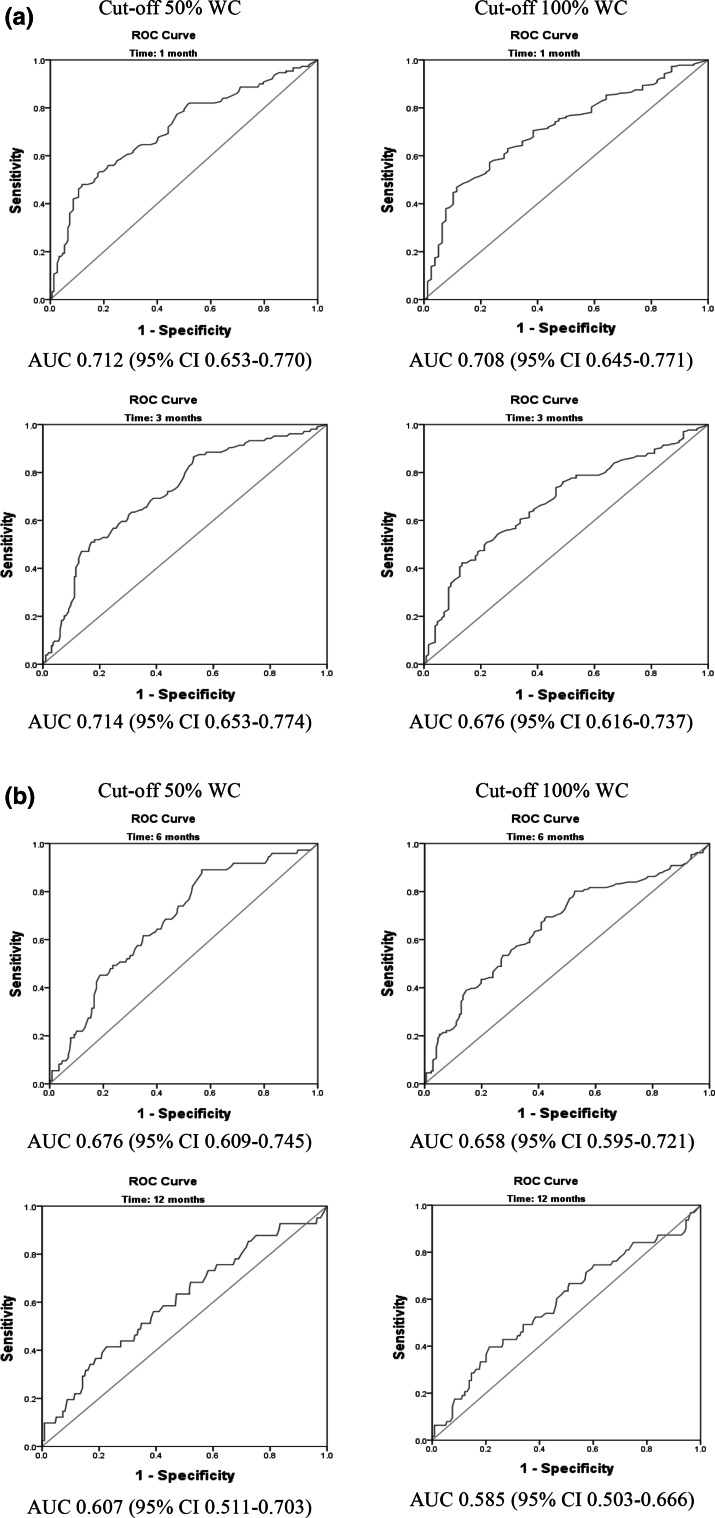
(AUC, Part 1). ROC curve of SFS total score at baseline with cut off values of work capacity 50 or 100 % at 1 month (*first row*) and 3 months (*second row*) follow-up to predict non return to work. (AUC, Part 2). ROC curve of SFS total score at baseline with cut off values of workcapacity 50 or 100 % at 6 months (*third row*) and 12 months (*fourth row*) follow-up to predict non return to work. *WC* workcapacity, *AUC* area under the curve, *CI* confidence interval

Sensitivity of the SFS scores transformed into DOT categories for N-RTW at 1, 3, 6 and 12 months ranged between and 0.37 and 0.98 when using the cut-off value of <50 % WC between 0.28 and 0.98, with the cut-off <100 % respectively (Table 2). Sensitivity was substantially higher in the DOT-transformed categories “light” to “very heavy” than in the “sedentary” to “minimal” categories (Table 2). The likelihood ratio for a positive test for N-RTW at 1, 3, 6 and 12 months decreases from 4.64 to 0.96 for the cut-off value <50 % WC, and from 4.32 to 0.79 for the cut-off value of <100 % WC. SFS score can be dichotomized into scores <100 and scores ≥100 points. Patients with scores <100 perceive themselves as having minimal working ability. With this dichotomized scores, Sensitivity for N-RTW with the cut-off of WC < 50 % ranged over time between 0.37 and 0.41, and specificity (=RTW) ranged between 0.80 and 0.92. For the cut-off of WC < 100 %: sensitivity for N-RTW ranged over time between 0.28 and 0.34 and specificity (=RTW) ranged between 0.81 and 0.94 (based on data in Table 2, separately available on request). All ROC curves are displayed in Fig. 3. The AUC reached the cut-off for “acceptable” (>0.70) only three out of eight times: at 1 month follow for both WC cut-offs and at 3 months for cut-off 50 % WC.

**Table 2:** Predictive validity of DOT-transformed SFS categories for non-return to work at 1, 3, 6, and 12 months of follow-up

DOT categories (SFS score adapted)	N-RTW	RTW	Sens	Spec	+PV	Lr+	N-RTW	RTW	Sens	Spec	+PV	Lr+
	WC-Cut-off: 0–49 %	WC-Cut-off: 50–100 %					WC-Cut-off: 0–99 %	WC-Cut-off: 100 %				
1 month follow-up												
Minimal (0–99)	55	12	0.37	0.92	0.82	4.64	62	5	0.28	0.94	0.93	4.32
Sedentary (100–124)	26	19	0.54	0.80	0.72	2.65	41	4	0.46	0.88	0.92	3.99
Light (125–164)	43	66	0.83	0.36	0.56	1.30	72	37	0.78	0.41	0.79	1.32
Medium (165–179)	13	26	0.91	0.19	0.53	1.13	25	14	0.89	0.23	0.77	1.16
Heavy (180–194)	9	20	0.97	0.06	0.51	1.03	19	10	0.98	0.10	0.76	1.09
Very heavy (195–200)	4	9					5	8				
3 months follow-up												
Minimal (0–99)	43	24	0.41	0.88	0.64	3.41	56	11	0.32	0.91	0.84	3.69
Sedentary (100–124)	17	28	0.58	0.74	0.54	2.20	29	16	0.49	0.79	0.81	2.28
Light (125–164)	33	76	0.89	0.35	0.42	1.38	55	54	0.80	0.36	0.67	1.25
Medium (165–179)	6	33	0.95	0.19	0.39	1.17	17	22	0.90	0.19	0.64	1.11
Heavy (180–194)	3	26	0.98	0.06	0.35	1.04	14	15	0.98	0.07	0.59	1.05
Very heavy (195–200)	2	11					4	9				
6 months follow-up												
Minimal (0–99)	28	39	0.38	0.83	0.42	2.25	45	22	0.34	0.87	0.67	2.67
Sedentary (100–124)	12	33	0.55	0.69	0.36	1.74	21	24	0.50	0.73	0.59	1.87
Light (125–164)	26	83	0.90	0.32	0.30	1.34	42	67	0.82	0.34	0.49	1.25
Medium (165–179)	4	35	0.96	0.17	0.27	1.16	8	31	0.66	0.16	0.45	0.79
Heavy (180–194)	1	28	0.97	0.05	0.25	1.02	10	19	0.96	0.05	0.44	1.01
Very heavy (195–200)	2	11					5	8				
12 months follow-up												
Minimal (0–99)	15	52	0.37	0.80	0.22	1.84	21	46	0.33	0.81	0.31	1.73
Sedentary (100–124)	6	39	0.51	0.65	0.19	1.47	10	35	0.49	0.66	0.28	1.45
Light (125–164)	13	96	0.83	0.28	0.15	1.16	19	90	0.79	0.28	0.23	1.11
Medium (165–179)	4	35	0.93	0.15	0.15	1.09	5	34	0.87	0.14	0.21	1.02
Heavy (180–194)	0	29	0.93	0.04	0.13	0.96	5	24	0.95	0.04	0.21	0.99
Very heavy (195–200)	3	10					3	10				

*N*-*RTW* not return to work based on the WC, *RTW* return to work based on the WC, *Spec* specificity, *Sens* sensitivity, +*PV* positive predictive value, *Lr*+ likelihood ratio of a positive test, *DOT* Dictionary of Occupational Titles, *SFS* Spinal Function Sort

## Discussion

The aim of the study was to extensively analyze measurement properties of the SFS in patients with WAD 6–12 weeks after injury. The majority (7 out of 8) of the a priori defined hypotheses for construct validity were not rejected. The SFS test structure was confirmed by a distinct factor loading. Test–retest reliability was good, however measure of error (LoA values) on an individual level were large relative to the scale range. Predictive validity of the SFS based on the AUC was acceptable in three out of 8 AUC: at 1 month for both cut-offs and at 3 months for cut-off 50 % WC. The SFS scores for the DOT-transformed categories “minimal” to “sedentary” workload were not able to identify those who will N-RTW (low sensitivity). The positive likelihood ratio for N-RTW was sufficient only for the categories “minimal” to “sedentary” for both cut-off WC < 50 % and WC < 100 %.

The SFS can, based on the measurement properties evaluated in this study, be recommended for clinical and research applications in patients in an occupational setting with sub-acute WAD and with different cultural backgrounds. Clinicians should be aware of the large measurement error of the SFS when making recommendations on individual level. The scores of the SFS may assist to predict N-RTW especially for medium, heavy and very heavy DOT categories. Application of the SFS may be a practical alternative or addition to other instruments with sufficient measurement properties. Practicality can be enhanced when half the items are removed. Further research should analyze if even more items can be removed (Cronbach’s α of half the SFS items is 0.97, indicating that item redundancy is still apparent).

The SFS scores in our sub-acute sample was substantially higher (mean 133 points, SD 42.7) than in two other validation studies with chronic low back pain patients in Europe (mean 105 points, SD 46.1), and in Australia (mean 116, SD 40.8) [5, 8]. A very high Cronbach’s α was found, which is in line with previous validation studies [5, 6, 8]. High internal consistency may be partly determined by a large number of items [30]. These high alpha values are indicative for item redundancy. In a sensitivity analysis we calculated Cronbach’s α and PCA values with half of the SFS items, with minimal changes in consistency and dimensionality. From a statistical point of view, half of the SFS items could be omitted, reducing the time requirement to fill out the questionnaire to 5 Min. (now, 10–15 min.). In agreement with previous studies, four items, with very heavy lifting tasks, could be removed without affecting the measurement properties of the SFS [5, 6]. Our results concerning reliability measured with ICC 0.80 are lower than two reliability studies 0.89 and 0.98 respectively [6, 8]. The LoA values found in a rehabilitation setting in the French-speaking area of Switzerland were ±11 while in the German-speaking area the values were ±27, whereas our results were ±33 [6]. In the studies of the German speaking sample the SFS was part of case-closure FCE setting to define fitness-for-work, whereas in the French-speaking sample this was not the case [5, 6]. One reason for the differences in reliability and agreement may be the difference in interval between test and retest; 2–3 days compared to 7 days in our study. Another reason may be that our patients were in a sub-acute stage of WAD which may change more on a daily basis compared to chronic patients. The ability to predict N-RTW in our study was substantially lower than in a sample of patients with CLBP [5] although follow-up times were similar. Albeit some similarities, the work rehabilitation setting and large proportion of blue collar workers with a Non-Swiss cultural background, several other reasons may explain these differences.

First, the proportion of patients who did N-RTW was substantially lower at 3 and 12 month follow-up in our study sample compared in patients with CLBP with rates between 34 and 16 %, and 62 and 54 % respectively. This may be due the fact that the CLBP patient had on average a significantly longer duration of 200 days off work, compared to 90 days in this study. Therefore, a smaller proportion of WAD patients is expected to N-RTW due to the benign natural course of the disorder despite perceived disability [31]. Further, we used WC data from the physician and the insurance. Moreover, legal regulations in Switzerland recently changed allowing to close claims of patients with WAD within the first 1 or 2 years which is not the case in CLBP [32]. These changes may have influenced N-RTW rates in patients with WAD which depend on the legal jurisdictions [33]. Hence, the validity of the SFS should be tested also in patients with WAD in other health cares systems. Secondly, in one study patients were classified as RTW if they had worked at least 1 day in the follow-up period [5]. These differences influence the proportion of patients classified as RTW or N-RTW, and therefore the results concerning the predictive properties of the SFS [34]. Third, the differences in symptoms of patients with WAD differ in part from those with CLBP. And forth, the depicted tasks of the SFS involving the spine may be perceived to the neck differently from the lower back.

Future studies should investigate whether a short version of the SFS would lead to similar measurement properties. Computer based measures could offer some advantages over a paper form. By using Item Response Theory (IRT) techniques only suitable items are assigned based on the response pattern of the evaluee. First results using a computer based measure similar to the SFS are promising, but need further evaluation in clinical samples [35, 36].

### Limitations

We used hypotheses and cut-off points based on the results of previous studies. These cut-offs may viewed as arbitrary. Moreover, we analysed WC in % which may lead to different results then compared to self-report of the employee, or other reporting measures [37–39]. Moreover, the psychometric properties of WC in % are unknown. WC may rely on physicians interpretations and patients report [40]. Finally, replication studies are needed because the results differ in other populations, contexts and FCE procedures.

## Conclusion

In patients with sub-acute WAD test–retest reliability, internal consistency, construct- and structural validity of the SFS were adequate. LoA was substantial. Sensitivity to accurately predict N-RTW was poor.

Based on the AUC the predictive validity of the SFS for N-RTW of patients with sub-acute WAD from an outpatient work rehabilitation setting was only sufficient for the short term.

## Abbreviations

SFS: Spinal Function Sort questionnaire
WAD: Whiplash-associated disorder
FCE: Functional capacity evaluation
WC: Work capacity
(N-)RTW: (Non-)return to work
DOT: Dictionary of Occupational Titles
